# Hard exudates in diabetic macular edema after intravitreal anti-VEGF therapy: a post-hoc analysis of the DRCR protocol T trial

**DOI:** 10.1007/s00417-025-07015-0

**Published:** 2025-11-08

**Authors:** Kangyeun Pak, Changki Yoon, Srinivas R. Sadda

**Affiliations:** 1 Doheny Eye Institute, 150 N. Orange Grove Blvd, Pasadena, CA 91103 USA; 2 Department of Ophthalmology, Inje University Haeundae Paik hospital, Pusan, Korea; 3 Department of Ophthalmology, David Geffen School of Medicine at UCLA, Los Angeles, CA USA; 4 Department of Ophthalmology, Seoul National University Hospital, Seoul, Korea

**Keywords:** Diabetic macular edema, Deep learning algorithm, Hard exudates

## Abstract

**Aim:**

To quantitatively analyze change in the extent of hard exudates (HEs) following anti-VEGF therapy for diabetic macular edema (DME) and its relationships with visual outcomes.

**Methods:**

This post-hoc analysis of DRCR Protocol T included 260 eyes of 260 patients. The volume of HEs was measured by automatically quantifying hyper-reflective foci (HRF) on structural optical coherence tomography (OCT) volumes using a supervised convolutional neural network architecture, “DUCK-Net”. HEs were quantified within the entire ETDRS grid as well as within the central subfield (CSF), inner ring (IR), and outer ring (OR) at baseline, 4, 12, 24, and 52 weeks (w) after treatment. The extent of HEs at baseline and over time was then correlated with visual acuity (VA) and retinal thickness outcomes.

**Results:**

Following initiation of anti-VEGF therapy, HEs significantly increased from baseline (0.0293 ± 0.0455 mm3) to w4 (0.0328 ± 0.0492 mm3) and peaked at w12 (0.0350 ± 0.0513 mm3), but decreased by w52 (0.0165 ± 0.0275mm3) within the entire ETDRS region (P = < 0.001, respectively), as well as within the OR and IR. Multiple regression analysis revealed that baseline HEs within the OR was one of the independent predictors of w52 VA (adjusted R2 = 0.160).

**Conclusions:**

Following anti-VEGF for DME, HEs initially increase, followed by a subsequent decrease over one year. Greater extent of HEs at baseline is associated with worse visual outcomes at one year.

**Supplementary Information:**

The online version contains supplementary material available at 10.1007/s00417-025-07015-0.

## Key Messages

**What is known:**

•The severity of hard exudates (HEs) in diabetic macular edema is deemed to be of visual significance.

**What is new:**

•After anti-VEGF therapy, HEs increase initially before decreasing with a more protracted increase in the outer portion compared to fovea in quantitative analysis.

•The extent of outer ring HEs was related with visual outcomes at 1 year.

**Supplementary Information:**

The online version contains supplementary material available at 10.1007/s00417-025-07015-0.

## Introduction

Diabetic retinopathy (DR) is an important vision-threatening sequela of diabetes mellitus (DM), with DME being the most common cause of vision loss [1]. Hard exudates (HEs), often accompanying DME, present as yellow-white deposits in the retina that are thought to consist of lipid-rich exudates. Although HEs can occasionally resolve in a spontaneous fashion, they can progress to form a fibrotic scar that can lead to severe vision loss [2]. Thus, the evaluation of HEs and their progression over time constitutes a topic of interest.

Despite the purported importance of this topic, however, studies about HEs are limited. One reason is that, unlike other ophthalmic assessments, no test providing direct quantitative or qualitative results exists. Early studies featured qualitative grading systems based on photographs [3]. Several quantitative methods used fundus photographs [4–7]. Recent reports have relied on structural optical coherence tomography (OCT), owing to its volumetric properties and ability to display the distribution of pathologic features across all retinal layers [8, 9]. More recently, Kim et al. reported that an automated method integrating information across all OCT B-scans within the volume scan could measure the volume of HEs and correlated well with area of HEs in fundus photography [10]. However, all of the aforementioned methods are tedious and required significant human intervention even when using 3rd party image analysis programs, which is not a practical approach for large-scale studies.

Indeed, recent advances of artificial intelligence (AI) in image analysis have proved to be remarkable. Deep learning algorithms are a novel AI-based approach which facilitate automated image analysis. Techniques and architectures such as convolutional neural networks are able to learn various patterns within images, enabling the model to identify specific images based on these learned features. To quantify HEs, there had been several attempts to apply deep learning algorithms to OCT images for segmentation of HEs [11–13].

The Protocol T trial, conducted by The Diabetic Retinopathy Clinical Research Network (DRCR.net), compared the efficacy of the three most commonly used anti-VEGF agents: ranibizumab, bevacizumab, and aflibercept on DME [14]. The imaging and other associated data collected in this trial have proven to be valuable for a number of significant post-hoc analyses [15, 16], though the impact of therapy on HEs has not yet been evaluated quantitatively.

In the present study, we used automated deep learning-based algorithms to measure the effect of intravitreal anti-VEGF injections on HEs in DME patients, and to correlate the extent of HEs with other ophthalmic parameters and vision over a period of 12 months.

## Method

This retrospective, longitudinal, Institutional Review Board-approved (University of California, Los Angeles, IRB#15–000083) study was performed in accordance with the Health Insurance Portability and Accountability Act and adhered to the tenets of the Declaration of Helsinki.

### Inclusion and exclusion criteria

We performed a post-hoc analysis of a multicenter randomized clinical trial, the DRCR.net Protocol T trial [NCT01627249]. A detailed description of the methods utilized for DRCR.net Protocol T has previously been published [14]. In brief, the participants were adults (≥ 18 years) with type 1 or type 2 diabetes who had at least one eye with a best-corrected Early Treatment Diabetic Retinopathy Study (ETDRS) visual acuity (VA) letter score of 78 through 24 (approximate Snellen equivalent, 20/32 − 20/320) and center-involved (CI) DME as determined by OCT. Patients were randomly assigned to aflibercept, bevacizumab, or ranibizumab in a 1:1:1 allocation.

For the present analysis, patients were included based on the following criteria: (1) patients with Cirrus OCT (Carl Zeiss Meditec, Dublin, CA) data and (2) patients who had follow-up OCT data at 4, 12, 24, and 52 weeks (w) after treatment, with no more than one missing visit among these timepoints. The exclusion criteria were as follows: (1) a signal strength ≤ 3; (2) poor image quality, for reasons other than low signal strength (e.g. poor centration, axial scan failed to capture the entire layer of retina); (3) any deformation of the retinal pigment epithelium (RPE) contour aside from small drusen (of note, we have observed that a smooth RPE contour is important for accurate automated segmentation); and (4) no HEs at baseline.

## Analysis of HEs using OCT

**Fig. 1:**
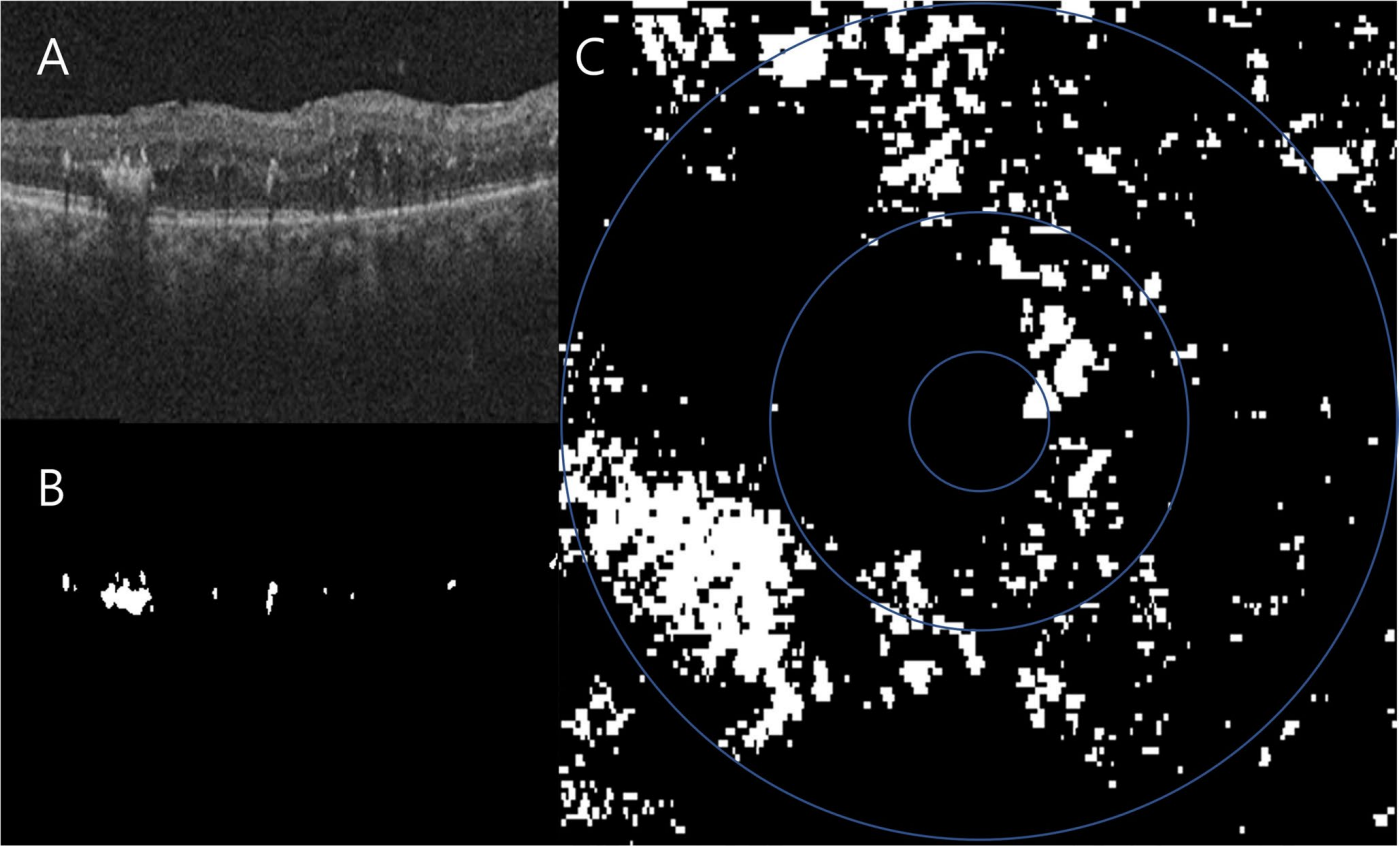
Quantification of Hyper-Reflective Foci Using optical coherence tomography (OCT) (**A**) The OCT B scan grayscale image was obtained. (**B**) The deep learning algorithm segmented hyper-reflective foci between the internal limiting membrane and retinal pigment epithelium. **C**: The Hard exudates projection map was generated and ETDRS grid was superimposed to divide the map into the central subfield, inner ring, and outer ring

The volume of HEs was measured using previously reported method [17]. In brief, using a 512 A-scan × 128 B-scan OCT volume (6 × 6 mm) with an axial dimension of 2 mm, the area of HEs was measured as total pixels of hyper-reflective foci (HRF) between the internal limiting membrane and RPE in each B-scan image (Fig. 1A, B). Only three or more contiguous pixels of hyperreflectivity in the B-scan were considered to be HRF for purposes of quantification [18]. A deep learning model was developed to segment HRF from each OCT B-scan. A modified U-Net architecture, “Duck-net”, was trained on manually segmented OCT B-scans of HRF. The dataset for training and testing of the model included 1811 OCT scans from 15 patients with macular edema from diabetic retinopathy or branch retinal vein occlusion. Five-fold cross-validation was used to optimize and evaluate model performance. From the overall set, 150 scans were randomly selected and left out of the model training process, and reserved for testing the model’s performance. The Dice coefficient and accuracy of the algorithm were 0.727 and 99.96%, respectively, on the test set. The number of automatically segmented pixels was summed across all of the 128 B-scans, generating a value that effectively represented a volume of HEs in voxels. Based on the known physical dimension of the voxel (633 × 422 × 128), the total volume of HEs could be computed in mm^3^. A projected image map of HEs was created and the ETDRS grid was superimposed to divide the map into the central subfield (CSF), inner ring (IR), and outer ring (OR; see Fig. 1C) regions. The volume of HEs was measured in the entire 6 × 6 mm scan (Total) and in each region.

## Data collection

The primary outcome measurement was the volume of HEs at baseline, 4, 12, 24, and 52w after treatment in total and each ETDRS region (CSF, IR, OR). Demographic and other clinical data were obtained from the DRCR.net database. Baseline variables that were considered included the VA letter score, central subfield macular thickness (CST), age, gender, type of DM, mean arterial blood pressure (BP), hemoglobin A_1c_ level, lens status, DM duration, body mass index, and prior laser history. The baseline characteristics except CST were compared between the analysis cohort, excluded cohort, and the entire protocol T cohort. CST could not be effectively compared as the entire cohort included more than one OCT device. The VA letter score and CST were assessed at each study timepoint. Changes in HEs, VA, and CST were also measured.

### Statistical analysis

Statistical analysis was conducted using SPSS (version 12 for Windows; SPSS Inc., Chicago, IL, USA). One-way Analysis of Variance and chi-square test were used to compare baseline variables with excluded and entire cohort of protocol T. Paired t-tests were used to compare the HEs volume from the baseline with each studied timepoint. Pearson’s correlation analysis was used to find other clinical variables associated with the extent of HEs and the change in HEs over time. Linear regression analysis was used to determine the factors that influenced VA and VA change. Potential related variables with a P-value of ≤ 0.10 on univariable analysis were included in the multivariable analysis. Stepwise regression was used for multivariable analysis and the model was adjusted so as not to infringe on multicollinearity. Data are presented as mean ± standard deviation (SD). For all tests, a value of *P* < 0.05 was considered statistically significant. A Bonferroni correction was applied for p-value adjustment to reduce the risk of type 1 error when performing multiple comparison.

## Results

Among 660 eyes in the original Protocol T trial, 260 eyes from 260 subjects (39.4%) met our criteria and were included in the present analysis. The average age of included subjects was 61.2 (± 10.2) years, with 130 subjects (50.0%) being female. The mean duration of DM was 16.8 (± 10.2) years. The VA letter score and CST at baseline were 65.8 (± 10.9) and 451.9 (± 130.9) um, respectively. The baseline variables were well-balanced when compared with excluded and entire cohort of protocol T (Table 1).

**Table 1:** Baseline characteristics comparing included, excluded group and protocol T trial

Variables	Included Cohort	Excluded Cohort	Entire Protocol T Cohort	*P*-value
Total number of eyes	260	400	660	
Age, years	61.2 ± 10.2, [32–87]	60.1 ± 10.5, [24–87]	60.5 ± 10.4, [24–87]	0.405
Sex (% of female), %	50.0	44.2	46.5	0.319
VA letter score	65.8 ± 10.9, [24–78]	64.2 ± 11.5, [27–78]	64.8 ± 11.3, [24–78]	0.182
Macular thickness, Um	451.9 ± 130.9, [290–944]	-	-	
Intraocular pressure, mmHg	15.6 ± 3.1 [6–25]	15.3 ± 3.3, [6–29]	15.4 ± 3.2, [6–29]	0.599
Mean arterial blood pressure, mmHg	101.4 ± 12.5, [71–131]	101.8 ± 12.8, [64–133]	101.6 ± 12.7, [64–133]	0.942
HbA1c	8.0 ± 1.7, [5.1–14.7]	8.0 ± 1.7, [4.9–15.9]	8.1 ± 1.7, [4.9–15.0]	0.571
Lens status (% of phakic), %	73.1	77.2	75.6	0.475
Duration of diabetes, years	16.8 ± 10.2, [0–55]	17.4 ± 10.8, [0–62]	17.1 ± 10.6, [0–62]	0.774
Prior anti VEGF treatment (% of yes), %	14.2	12.1	12.9	0.757
Body mass index, kg/m²	33.6 ± 7.8, [20.6–64.0]	33.7 ± 7.8, [18.4–68.1]	33.7 ± 7.8, [18.2–68.1]	0.974
Prior PRP (% of yes), %	11.5	19.3	16.2	0.052
Prior focal laser (% of yes), %	34.6	38.6	37.0	0.875

* Values were presented as mean ± standard deviation, [range]

*HEs* hard exudates, *VA* Early treatment diabetic retinopathy study visual acuity, *VEGF* vascular endothelial growth factor, *PRP* Pan-retinal photocoagulation

### Change in HEs over time

**Fig. 2:**
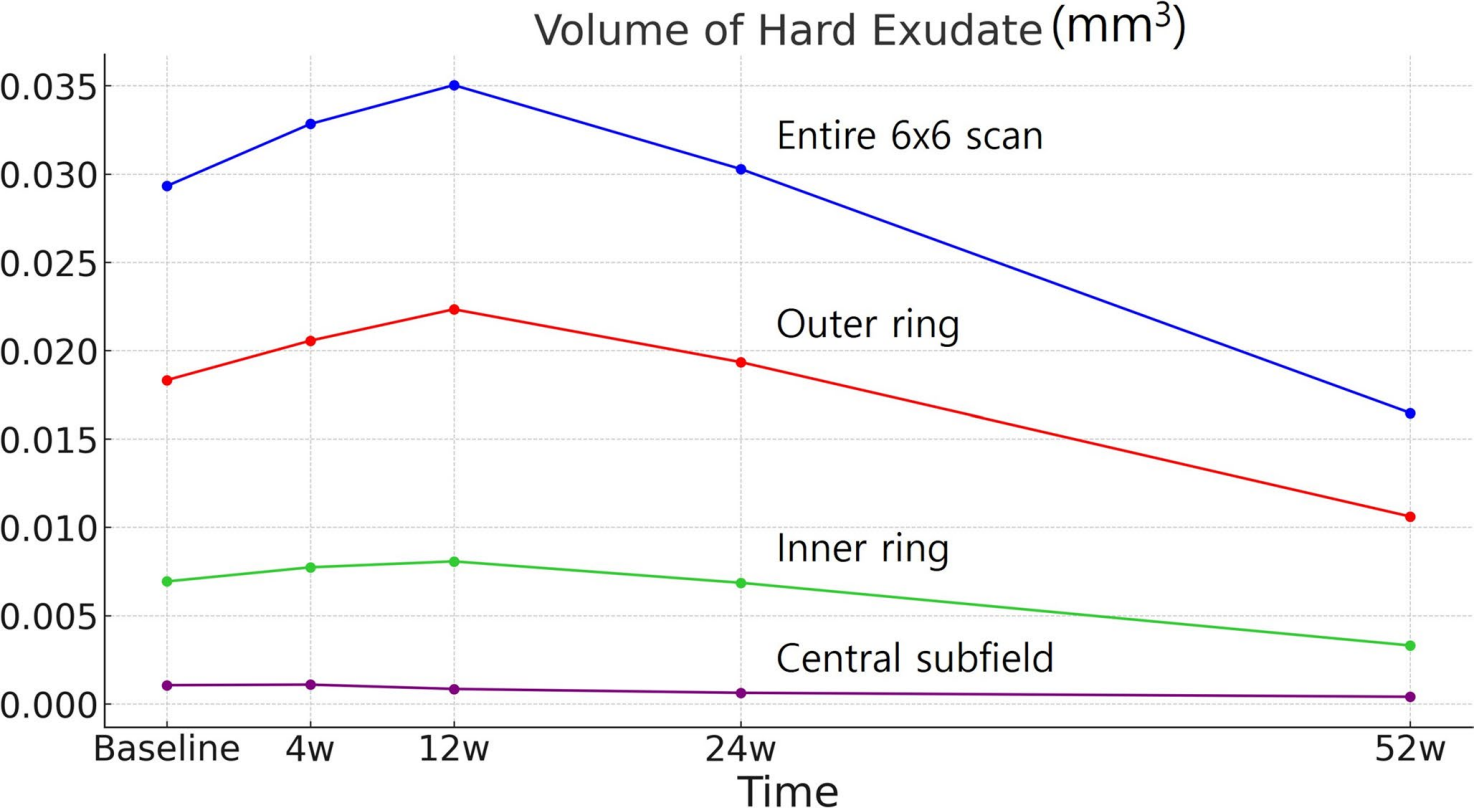
Time Course of Change in Hard Exudates (HEs) Volume by Region. While the HEs volume does not increase before declining in the central subfield, it increases and peaks at 12 weeks in the inner and outer rings. The HEs volume is substantially greater in the outer ring and thus the entire macula HEs trajectory behaves similarly

Total HEs volume increased significantly from baseline (0.0293 ± 0.0455 mm^3^) to w4 (0.0328 ± 0.0492 mm^3^), peaked at w12 (0.0350 ± 0.0513mm^3^), and decreased at w52 (0.0165 ± 0.0274mm^3^) (p = < 0.001, < 0.001, and < 0.001, respectively). HEs in the OR and IR increased from baseline to w4 (p = < 0.001, < 0.001, respectively), peaked at w12 (p = < 0.001, 0.003, respectively), and decreased at w52 (p = < 0.001, < 0.001, respectively). CSF HEs significantly decreased at w24 and w52 (*p* = 0.001, < 0.001, respectively; see Table 2; Fig. 2 and Figure S1). By w52, none of the subjects showed complete resolution of HEs when considering the total region. Complete resolution was observed for 76 eyes (29.2%) in the CSF, 10 eyes in the IR (3.8%), and 1 eye (0.4%) in the OR.

**Table 2:** Change in HEs from baseline to 4, 12, 24, and 52 weeks within entire 6 × 6 mm Scan, central Subfield, inner ring, and outer ring

	Volume of HEs (Mean ± SD, mm^3^), *p*-value			
	**Entire 6 × 6 mm scan**	**Central subfield**	**Inner ring**	**Outer ring**
Baseline	0.0293 ± 0.0455	0.0011 ± 0.0028	0.0069 ± 0.0124	0.0183 ± 0.0310
4 weeks	0.0328 ± 0.0492 (111.9)	0.0011 ± 0.0027 (100)	0.0077 ± 0.0127 (111.6)	0.0206 ± 0.0333 (112.6)
12 weeks	0.0350 ± 0.0513 (119.5)	0.0008 ± 0.0023 (72.7)	0.0081 ± 0.0137 (117.4)	0.0223 ± 0.0346 (121.9)
24 weeks	0.0303 ± 0.0457 (103.4)	0.0006 ± 0.0018 (54.5)	0.0069 ± 0.0112 (100)	0.0193 ± 0.0311 (105.5)
52 weeks	0.0165 ± 0.0275 (56.3)	0.0004 ± 0.0012 (36.4)	0.0033 ± 0.0066 (47.8)	0.0106 ± 0.0183 (57.9)

HEs: Hard exudates; SD: Standard deviation

*The number in parentheses is percentage compared to baseline

.

## Association between HEs and other clinical variables

Total HEs at baseline showed a correlation with age (*r* = −0.158, *p* = 0.011), Hb1Ac (*r* = 0.198, *p* = 0.001), and DM duration (*r* = −0.221, *p* < 0.001). HEs in the OR and IR were also correlated with age (*r* = −0.138, −0.184, *p* = 0.026, 0.003, respectively), Hb1Ac age (*r* = 0.175, 0.177, *p* = 0.005, 0.004, respectively), and DM duration (*r* = −0.209, −0.140, *p* = 0.001, 0.024, respectively). CSF HEs was correlated with HbA1c (*r* = 0.157, *p* = 0.011) and DM duration (*r* = −0.140, *p* = 0.024). At baseline, CST was correlated with total HEs (*r* = 0.199, *p* = 0.001), IR HEs (*r* = 0.155, *p* = 0.012), and OR HEs (*r* = 0.199, *p* = 0.001). Similarly, the change in CST showed a correlation with the change in HEs. At w52, the change in CST was positively correlated with change in total HEs (*r* = 0.254, *p* < 0.001), IR HEs (*r* = 0.206, *p* = 0.001), and OR HEs (*r* = 0.252, p = < 0.001). In contrast, at w4, the change in CST was negatively correlated with the change in total HEs (*r* = −0.192, *p* = 0.003), and OR HEs (*r* = −0.188, *p* = 0.004).

## Visual acuity and other clinical variables

Univariable analysis revealed that VA at w52 was associated with the CST at all studied timepoint, change in CST at w4, age, HEs at selected timepoint within selected region, change in HEs at selected timepoint within selected region, and lens status. Multivariable analysis, however, demonstrated that the only independent predictors of VA at w52 were CST at 52w (adjusted beta = −0.300, p = < 0.001, partial R^2^ = 0.072), baseline OR HEs (adjusted beta = −0.280, p = < 0.001, partial R^2^ = 0.072) and pseudophakic lens status (adjusted beta = −0.153, *p* = 0.019, partial R^2^ = 0.025, adjusted R^2^ = 0.160). Details of the univariable and multivariable analyses are provided in Table 3 and Table S1. With regard to the change in VA from baseline to w52, CST at selected timepoint, change in CST at all timepoints, age, mean arterial BP, duration of DM, and CSF HEs at w24 and w52 were significant predictors identified in the univariable analysis. In the multivariable analysis, change in CST at w52 (adjusted beta = −0.423, p = < 0.001, partial R^2^ = 0.142), CST at w12 (adjusted beta = −0.199, *p* = 0.002, partial R^2^ = 0.043) and DM duration (adjusted beta = −0.159, *p* = 0.014, partial R^2^ = 0.026) were the only independent predictors (adjusted R^2^ = 0.259; see Table 3 and Table S2).

**Table 3:** Factors affecting visual acuity at 1 year and change in visual acuity from baseline to 1 year in multivariable analysis

valuables	Standarized beta	*P* value	Partial *R*^2^
Visual acuity at 1 year (Adjusted R^2^ = 0.160)			
CST at w52	−0.300	< 0.001	0.072
OR HEs at baseline	−0.280	< 0.001	0.072
Pseudophakic lens	−0.153	0.019	0.025
Change in visual acuity from baseline to 1 year (Adjusted R^2^ = 0.259)			
Change in CST at w52	−0.423	< 0.001	0.142
CST at w12	−0.199	0.002	0.043
Duration of diabetes	−0.159	0.014	0.026

*In univariate analysis, only statistically significant factors were presented

*Only valuables with *P* < 0.10 went on to the multivariate analysis

*CST* central subfield macular thickness, *HEs* hard exudates, *IR* inner ring, *OR,* outer ring, *CSF* central subfield

## Discussion

In this post-hoc analysis of a subcohort of individuals from DRCR.net Protocol T, we analyzed the volume of HEs over time after anti-VEGF therapy as quantified using a deep learning algorithm. HEs increased in the first four weeks after the start of treatment followed by a significant decrease over the following year. However, the time course differed topographically. For example, the CSF showed faster absorption of HEs compared to the outer portions of the macula. At 4 weeks following the start of anti-VEGF therapy, HEs were noted to increase while CST dramatically decreased, presumably related to the precipitation of additional lipid as the fluid component of the exudate was resorbed following treatment.

The most commonly used indicator of disease activity and treatment efficacy in the setting of DME is CST, which reflects the amount of accumulated fluid in the fovea [19]. CST is particularly useful clinically as it is readily provided by most OCT devices and may serve as an easy way to track treatment response over time. CST has also been shown to be associated with visual acuity [20], with greater levels of edema generally associated with poorer visual acuities. This association, however, has been modest at best, and is one reason why anatomic OCT measures have not been accepted as primary clinical trial endpoints. For example, studies have shown that VA can deteriorate even while the CST remains in the normal range, and VA can improve in spite of an increase in CST [20]. This is because many other OCT parameters, including the status of the outer retinal bands (i.e. the photoreceptors) and disorganization of the inner retinal layers (DRIL) due to ischemia, can impact visual acuity [21–24]. Our study is overall consistent with these prior reports, as the CST was a significant predictor of w52 VA in our cohort, but only explained a small portion of the variability with a partial R^2^ of 0.072. Of note, the CST at w12 and change in CST at w52 were modest but significant predictors of change in VA at w52. Specifically, a lower CST at w12 and a greater reduction in CST at w52 were associated with a greater increase in VA at w52,

In addition to water accumulation from retinal vascular hyperpermeability in DME, HEs are considered one of the most important markers for retinal edema and are frequently observed in these eyes [20]. Indeed, prior to OCT, HEs on exam were commonly used as a surrogate to alert the clinician to the presence of macular edema. HEs were also deemed to be of visual significance as they can lead to photoreceptor and neuronal degeneration in the outer plexiform layer [3]. Importantly, an increase in the number of HEs in patients with DME, particular when located closer to the foveal center, has been associated with an increased risk of visual loss and subretinal fibrosis [2, 3].

Reduction in HEs following intravitreal anti-VEGF therapy has been observed, and several previous reports have used color fundus photographs for quantification and monitoring of HEs over time [25–27]. While color fundus photograph has some advantages in that HEs can be distinguished from other opacities owing to their yellow color, it is limited compared to OCT, due to its lack of depth resolution [8]. In contrast, OCT may not only be more sensitive for detecting these HEs, but also provides three-dimensional information regarding the extent and distribution of HEs throughout the retinal layers [28]. A limitation of OCT is that not all HRF may represent HEs. Indeed, some HRF may represent inflammatory cells or other materials, which may also be important biomarkers of treatment response [29]. Generally, investigators have used a size criterion in an attempt to separate HEs from other components, with HEs typically assumed to be larger in size [30]. This separation, however, is likely to be imperfect. We used a size criteria of 3 contiguous pixels based on prior reports, but we must acknowledge that our HEs volume likely included some HRF that were not lipid, and also missed some tiny bits of lipid that fell below our size criterion. While this is a limitation of any such analysis, it does not preclude the parameter from serving as a useful biomarker.

Prior OCT studies using an en face approach have demonstrated the efficacy of anti-VEGF therapy for resolving HEs [9]. En face approaches will typically obtain a slab spanning from the ganglion cell layer to the external limiting membrane, and quantify all hyperreflective lesions within that slab. The problem with an en face approach, is similar to that of quantification using color photographs, as the axial depth information is lost, and as there may be HEs at multiple depths along the same A-scan, the en face measurements will generally be underestimates of the true HEs burden. We demonstrated this underestimation to be the case in a prior study, where HEs were manually segmented on every B-scan for quantification, and compared to the result from the en face strategy. ^8^. Manual segmentation of HEs on all B-scans within a volume, however, is tedious and exhaustive and not practical for large-scale studies. Thus, in the present analysis we employed a deep learning algorithm for automatic identification and quantification of HEs from all B-scans in the volume. We should note that other groups have also developed deep learning algorithms for HE quantification. Xie et al. ^12^ modified 3D-UNet for HRF segmentation and demonstrated its utility in 27 patients. Yao et al. ^11^ also modified U-Net for HRF quantification and evaluated its performance in 112 B-scans. These small studies, however, mostly focused on algorithm performance and not on the clinical significance of HEs quantification.

In our analysis, we did observe a significant and substantial reduction in HEs from baseline to w52 of 43.7% (from a volume of 0.0293mm [3] at baseline to 0.0165mm [3] at w52 within the entire 6 × 6 scan region). Notably, even at w52, none of subjects showed complete resolution of HEs in the entire macula, though a small portion of subjects did show complete resolution within CSF (29.2%) and IR (3.8%). This qualitative result was inconsistent with the marked quantitative reduction of 43.7%, however, was consistent in that slower absorption occurred in outer macula. Of note, HEs increased early on before ultimately decreasing. This is consistent with our previous report [31] that HEs tend to increase 2 months following the initiation of intravitreal ranibizumab therapy, though the increase was not statistically not significant. In a study using the dexamethasone implant (Ozurdex, Allergan, Irvine, California, USA), HEs also tended to increase and central HEs increased significantly at 1 month [32]. The authors hypothesized that the rapid resolution of the fluid component of macular edema with a relatively lagged absorption of lipid could make HEs more distinctively visible [32]. Our observation of a negative correlation between the change in HEs and CST at w4 is consistent with this notion.

The delayed clearance of HEs is perhaps not surprising given that removal of lipid from the retina is thought to require the activity of phagocytic inflammatory cells [33]. As interesting observation from our analysis is the topographic variation in the trajectory of HEs evolution. Whereas the HEs never increased in CSF, they continued to increase until w12 in the inner and outer rings prior to declining. This was despite the fact that all subjects in the study were required to have CI edema. This observation suggests that there could be different mechanisms or efficiencies of lipid clearing in the central vs. outer regions of the macula Given the requirement for phagocytic cells to participate in lipid clearance, regional variations in the density or functional activity of these cells may exist across the macula. Furthermore, if these cells predominantly originate from the choroidal circulation, the relatively thinner foveal region could provide a shorter diffusion pathway, potentially facilitating more rapid access of these cells to lipid exudates after the resolution of the fluid component. Further studies such as the difference in the clearance rate of inner and outer retina (shorter pathway from choroid in inner retina) will be required, however, to elucidate these differential mechanisms.

Several baseline parameters were related to the baseline extents of HEs, with HbA1c showing a positive correlation, while age and DM duration showed a negative correlation. HbA1c is a previously known associated risk factor for HEs [3]. Uncontrolled BP is also a previously reported risk factors for HEs, but interestingly were not significantly correlated in the present study. It is interesting that older subjects and subjects with a greater duration of DM had a lower baseline HEs level. We do know that HEs also resolve spontaneously [2], and it is possible that patients with longer duration of DM may have developed adaptive mechanisms that may have facilitated their clearance. We acknowledge, however, that we do not have a satisfactory explanation for this observation and further research on this topic is needed.

The correlation between VA and HEs was perhaps the most interesting aspect of our analysis. Prior reports from the ETDRS have highlighted that greater extent of lipid exudates was associated with worse visual outcomes [2, 3], though that was in an era of focal laser photocoagulation. Focal laser photocoagulation is generally associated with slower resolution of edema compared to anti-VEGF therapy [34]. In the present analysis in the setting of anti-VEGF therapy, the extent of HEs in the OR at baseline were a significant independent predictor of w52 VA. While the correlation was admittedly modest, it should be noted that this was the only *baseline* OCT parameter that was predictive of w52 VA. Notably, baseline CST was not an independent predictor of w52 vision nor visual gain at w52. We note, however, that the explanatory power of regression model is modest. Inability to measure known variables including OCT parameters (e.g., DRIL, status of outer retinal band) is thought to be the reason.

It was interesting that the outer macula was predictive but not the central subfield. We speculate that this may be related to our observation that HEs tended to clear more slowly in the outer ring compared to central subfield, but it will be important to determine if this finding replicates in future studies. Overall, our findings suggest that even in the anti-VEGF era, baseline HEs, particularly in the outer macula, may have some prognostic relevance for VA outcomes.

Our study does have several limitations which should be considered when assessing our findings. First, this is a post-hoc analysis and the original study was not designed or powered to address questions related to HEs. Second, our study is susceptible to selection bias as we could only consider about 40% of the subjects originally enrolled in Protocol T trial as we required Cirrus OCT data. The reason we limited our study to subjects with Cirrus OCT data was related to scan density. With 128 B-scans over 6 mm, the interscan spacing for the Cirrus is < 50 microns. For the Spectralis OCT, which accounted for the most of the remainder of the cohort, the acquisition protocol only required 47 B-scans, resulting in an interscan spacing of >120 microns. Given the small size of HE lesions, we were concerned that HE volumes from the Spectralis would be significantly underestimated and not comparable to the Cirrus OCT. However, we should note that even with the denser Cirrus OCT volumes, there is still 47 ㎛ between B scans, and thus errors from interpolation may still occur. Despite only including the Cirrus OCT subset, we did confirm that the baseline characteristics were well-balanced compared to the total cohort. A third potential limitation is that we also included images with a lower signal strength than is generally accepted. For example, in many clinical trials, a signal strength ≥ 7 is required for reliable analysis at the reading center. We must acknowledge that HEs quantification in lower quality scans is more likely to have inaccuracies although we expect that deep learning approaches can partially compensate for this problem. A fourth limitation, as previously noted, is that we were not able to definitively differentiate HEs from other potentially bright structures. For example, small HRF might represent aggregates of activated microglial cells or migrated RPE cells [35, 36], though migrated RPE cells are less likely to be present in eyes with DME [36]. Fifth, AI-based deep learning algorithms while rapid and generally reliable, can certainly produce false-positive and false-negative results. Supervised convolutional neural networks such as our “DUCK-Net” algorithm, however, generally minimize these errors using a unique encoder-decoder structure with a residual down-sampling mechanism, in contrast to previously reported approaches [37]. A final limitation is that we only evaluated outcomes through one year, and thus cannot determine whether HEs continue to evolve in subsequent years or how baseline HEs can influence long-term visual outcomes. These may be important questions for future analyses. Despite these limitations, our analysis has many strengths including use of data collected according to standardized acquisition protocol from a clinical trial as well as good characterization of the cohort with regards to demographics and other clinical data.

In conclusion, our study highlights that anti-VEGF therapy can be effective for reducing HEs in patients with CI DME, but the time course is different by region and when compared to the resolution of retinal thickening. Although retinal thickening decreases rapidly with anti-VEGF therapy, HEs increase initially before decreasing, with a more protracted increase in the outer portions of the macula compared to the fovea. The extent of HEs, particularly in the outer ring of the macula, appear to be relevant for visual outcomes at one year following the initiation of therapy.

## Supplementary Information

Below is the link to the electronic supplementary material.

Supplementary Material 1 (DOCX 219 KB)
