# Supplementary material for: Hard exudates in diabetic macular edema after intravitreal anti-VEGF therapy: a post-hoc analysis of the DRCR protocol T trial
Source: Graefes Arch Clin Exp Ophthalmol. 2025 Nov 8;264(2):427–36. doi: 10.1007/s00417-025-07015-0 (PMC12923397; doi:10.1007/s00417-025-07015-0)
Supplement: Supplementary file 1 — Supplementary Material 1 (DOCX 219 KB) [file 417_2025_7015_MOESM1_ESM.docx]

Table S1. Factors affecting visual acuity at 1 year in univariable analysis.

|  | Univariate analysis | | |
| --- | --- | --- | --- |
| valuables | **beta** | **P value** | **R2** |
| CST at baseline, w4, w12, w24, and w52 | -0.016, -0.024, -0.014, -0.022, -0.030 | 0.001, 0.001, 0.055, 0.003, <0.001 | 0.039, 0.042, 0.015, 0.036, 0.073 |
| Chage of CST at w4 | 0.012 | 0.013 | 0.025 |
| Age | -0.190 | 0.003 | 0.033 |
| Pseudophakic lens | -3.178 | 0.034 | 0.017 |
| Total HEs at baseline, w4, w12, w24, and w52 | -55.6, -46.0,  -42.4, -44.0,  -81.6 | <0.001, 0.001, 0.001, 0.003, 0.001 | 0.052, 0.044, 0.044, 0.035, 0.043 |
| CSF HEs at w52 | -994.3 | 0.069 | 0.013 |
| IR HEs at baseline, w4 | -106.1, -107.3 | 0.048, 0.041 | 0.015, 0.016 |
| OR HEs at baseline, w4, w12, w24, and w52 | -85.3, -72.4,  -67.7, -65.8,  -124.3 | <0.001, 0.001, 0.001, 0.003, 0.001 | 0.061, 0.050, 0.051, 0.036, 0.045 |
| Change in Total HEs at w52 | 46.1 | 0.018 | 0.022 |
| Change in IR HEs at w24 | 134.6 | 0.089 | 0.012 |
| Change in OR HEs at w24, and w52 | 62.9, 75.0 | 0.077, 0.009 | 0.013, 0.026 |

CST: central subfield macular thickness; HEs: hard exudates; IR: inner ring, OR; outer ring; CSF: central subfield.

Table S2. Factors affecting change in visual acuity from baseline to 1 year in univariable analysis.

|  | Univariable analysis | | |
| --- | --- | --- | --- |
| valuables | **beta** | **P value** | **R2** |
| CST at baseline, w12, w24, and w52 | 0.016, -0.016, -0.022, -0.026 | <0.001, 0.010, <0.001, <0.001 | 0.050, 0.026, 0.050, 0.075 |
| Change in CST at w4, w12, w24, and w52 | -0.025, -0.019, -0.025, -0.027 | <0.001, <0.001, <0.001, <0.001, | 0.111, 0.082, 0.143, 0.163 |
| Age | -0.174 | 0.002 | 0.038 |
| Mean arterial blood pressure | 0.088 | 0.051 | 0.015 |
| Duration of diabetes | -0.177 | 0.001 | 0.039 |
| CSF HEs at w24, w52 | -681.7, -838.6 | 0.034, 0.070 | 0.018. 0.013 |

CST: central subfield macular thickness;

**Figure S1.** Overall Time Course of Hard Exudates (HEs) at Each Visit According to Region.


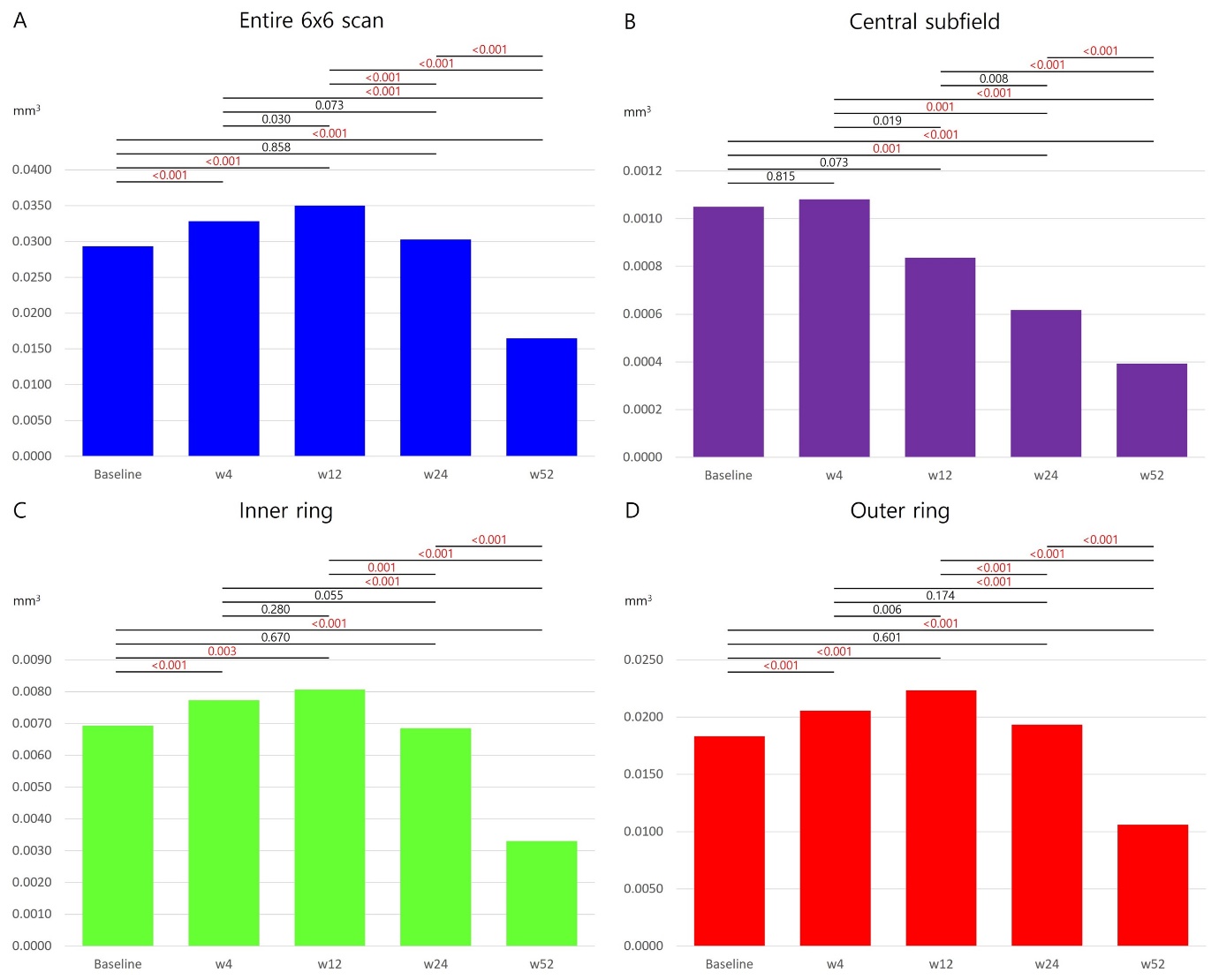


Bar graphs show HEs volumes at each time point. P-values as shown are from pairwise comparisons between the bars as indicated by the line below each P-value.
